# A Retrospective Cohort Study Comparing Robot-Assisted and Conventional Fluoroscopy-Guided Pedicle Screw Placement

**DOI:** 10.3390/jcm14196831

**Published:** 2025-09-26

**Authors:** Hassan Seif, Emanuele Maragno, Marco Gallus, Szabolcs Szeöke, Michael Schwake

**Affiliations:** 1 Department of Spine Surgery, Brüder St. Josef Hospital, D-33098 Paderborn, Germany; h.seif@bbtgruppe.de (H.S.); s.szeoeke@bbtgruppe.de (S.S.); 2 Department of Neurosurgery, Klinikum Dortmund, D-44145 Dortmund, Germany; emanuele.maragno@klinikumdo.de; 3 Faculty of Health, University Witten/Herdecke, D-58455 Witten, Germany; 4 Department of Neurosurgery, University Hospital Münster, D-48149 Münster, Germany; marco.gallus@ukmuenster.de; 5 Department of Neurosurgery, University of California San Francisco, San Francisco, CA 94143, USA

**Keywords:** spinal fusion, Robotics, navigation

## Abstract

**Background/Objectives**: Pedicle screw placement is crucial for restoring stability. Emerging robot-assisted technologies may offer enhanced precision and reduced radiation exposure. This study aimed to compare the accuracy and clinical outcomes of robot-assisted versus conventional fluoroscopy-guided pedicle screw placements. **Methods**: This retrospective cohort study included 218 patients undergoing pedicle screw placement at a single spine center between 2019 and 2023. Of these, 105 patients underwent robot-assisted surgery using the Mazor X™ Stealth Edition (Medtronic, Minneapolis, MN, USA), and 113 underwent conventional fluoroscopy-guided surgery. The primary outcome was screw placement accuracy (Grade 0 = optimal, Grades 1–3 = suboptimal, according to the Gertzbein–Robbins classification). Secondary outcomes included estimated blood loss (EBL), radiation exposure, length of hospital stay (LOS), clinical outcome according to the Macnab classification, postoperative pain, and adverse events. **Results**: Robot-assisted surgery demonstrated significantly higher accuracy in screw placement, with 93.33% achieving Grade 0 accuracy versus 78.76% in the conventional group (*p* = 0.002). This corresponded to an odds ratio (OR) of 3.78 (95% CI: 1.55–9.19, *p* = 0.003). The number needed to treat (NNT) to achieve one additional optimal screw placement was 6.9. Robot-assisted surgery demonstrated significantly higher accuracy in screw placement. Moreover, robot-assisted procedures were associated with reduced estimated blood loss (EBL), shorter length of stay (LOS), and lower radiation exposure times; patient-reported outcomes (VAS and Macnab) were also improved (OR = 3.34, 95% CI: 1.89–5.91). Duration of surgery, adverse events, and revision rates were comparable between the two groups. **Conclusions**: This study supports the clinical benefits of robot-assisted pedicle screw placement, particularly in achieving higher accuracy and reducing EBL and LOS. Future research should explore long-term outcomes, cost-effectiveness, and the generalizability of these results to a broader patient population.

## 1. Introduction

Pedicle screw fixation is a cornerstone of spinal surgery, employed to restore spinal stability following trauma, tumors, or degenerative conditions [1]. Traditionally, pedicle screw placement has relied on freehand techniques or fluoroscopic guidance, which, despite their widespread use, present challenges in achieving high accuracy. The thoracic spine, with its smaller pedicles and proximity to vital neural structures, poses particular challenges, as screw misplacement can lead to severe complications, such as neurological injury or screw loosening, ultimately compromising patient outcomes [2].

In recent years, advances in surgical technology have introduced several methods aimed at improving pedicle screw placement accuracy. Among these, robot-assisted technology has emerged as a promising solution, offering precision through real-time imaging, intraoperative guidance, and robotic-arm-assisted positioning [3,4,5,6]. Robotic systems integrate surgical planning workstations, intraoperative imaging, and control software, enabling continuous trajectory verification and minimizing risks of misplacement, even in minimally invasive procedures [4,7].

Robot-assisted pedicle screw placement not only enhances precision but also standardizes techniques, reducing outcome variability and aiding less experienced surgeons in achieving consistent results [8,9]. Additionally, robotic systems reduce fluoroscopy use, thereby minimizing radiation exposure for both patients and surgical teams, a significant advantage over traditional methods [3,10]. Enhanced visualization provided by robotic systems is particularly beneficial in anatomically complex cases, aligning with the growing demand for spinal procedures [11]. Potential limitations, such as increased risks of infection and hematoma, have been reported in the literature [12,13], though these were not statistically significant in our cohort.

Conversely, freehand techniques guided by fluoroscopy may lower infection risks and utilize tactile feedback, but they remain prone to errors stemming from surgeon fatigue and higher radiation exposure [7,10]. These trade-offs underscore the need for a systematic comparison of robotic-assisted and conventional freehand methods.

This study aims to compare the clinical and surgical outcomes of robot-assisted and conventional freehand pedicle screw placement. Key parameters include placement accuracy, complication rates, estimated blood loss, length of hospital stays, and infection rates. Furthermore, we aimed to investigate whether robotic guidance may reduce irradiation time in the operating room, minimizing the cumulative exposure of the surgical team. By elucidating the strengths and limitations of each approach, this study seeks to provide evidence to guide healthcare providers in selecting the most appropriate method based on patient needs, institutional capabilities, and surgeon expertise.

## 2. Materials and Methods

This retrospective cohort study was conducted to compare robot-assisted and conventional fluoroscopy-guided pedicle screw placement in spinal surgery. Data were collected from a single medical center between 01/2019 and 12/2023. Data were retrospectively extracted from the hospital’s electronic medical records, including surgery reports, anesthesia protocol radiographic assessments, and postoperative follow-up notes. The study was conducted according to the Declaration of Helsinki and approved by the institutional review board (2024-214-f-S).

### 2.1. Eligibility Criteria

Eligible participants included all consecutive patients aged 18 years and older who underwent pedicle screw instrumentation between January 2019 and December 2023 were screened from the institutional surgical database. Patients were included if they underwent instrumentation for osteoporotic or traumatic vertebral fractures or for degenerative conditions, including degenerative disk disease, spinal canal stenosis, or degenerative spondylolisthesis (Table 1). The decision to perform instrumentation was based on the treating surgeons’ clinical judgment, in accordance with institutional protocols, international guidelines for trauma, and established practice for degenerative conditions. Patients were categorized into two groups based on the surgical technique employed: robot-assisted or conventional fluoroscopy-guided pedicle screw placement. Patients under the age of 18 were excluded from the study. The allocation to each treatment group dependent on logistical reasons and resource availability at time of surgery. All surgeries were performed by, or under the supervision of two experienced surgeons HS and SS.

### 2.2. Surgical Methods

In the first cohort, percutaneous, fluoroscopic-guided screw insertion technique was employed. The procedure began with the insertion of a Jamshidi needle to establish the pedicle trajectory, under fluoroscopic X-ray guidance (anteroposterior and lateral views) throughout the surgery to ensure accurate screw placement. In the second cohort, a robotic-assisted technique utilizing the Mazor X™ Stealth Edition (Medtronic, Minneapolis, MN, USA) was implemented. Preoperative imaging, including fluoroscopic X-rays and computed tomography (CT), was processed with specialized software on the Mazor X workstation. This enabled three-dimensional anatomical reconstruction, pedicle measurements, trajectory optimization, and implant selection. The finalized surgical plan was then transferred to the robotic system, which facilitated precise screw placement during the procedure, which was conducted in accordance with the manufacturer’s guidelines, in a percutaneous and minimally invasive fashion.

### 2.3. Data Collection and Outcomes

The primary outcome of this study was the accuracy of pedicle screw placement, assessed using the Gertzbein–Robbins classification system [14]. In this classification system, Grade 0 indicates full containment within the pedicle with no cortical breach and was considered optimal placement. Grades 1–3 indicate breaches of increasing severity and were collectively categorized as suboptimal placements. Accuracy grading was independently assessed by two authors (HS, MS), with a third author (EM) resolving discrepancies.

The accuracy grade for each surgical case was determined by the highest screw grade observed, since even a single malpositioned screw may compromise stability or cause neurological complications requiring revision, this approach reflects the clinically relevant worst-case scenario for each patient.

Because we assume that experienced surgeons would place pedicle screw very accurately [15,16] and we expected an additional value of robotic-assisted surgery, we compared Grade 0 placements–defined as optimal placement–to Grade 1–3 placements–defined as suboptimal placement.

Secondary outcome variables included estimated blood loss (EBL), length of hospital stay (LOS), infection rates, hematoma occurrence, revision rates, and postoperative pain levels assessed by both the Visual Analog Scale (VAS) for pain intensity and the Macnab classification for overall functional improvement and patient satisfaction [17].

### 2.4. Risk of Bias

Potential sources of bias were addressed by applying consistent eligibility criteria for all participants and reviewing radiological data uniformly. Additionally, selection bias was potentially minimized by using data from a single center, where the surgical approach—robot-assisted or conventional—was determined based on standard clinical practice rather than random assignment.

### 2.5. Statistical Methods

Continuous variables were expressed as mean ± standard deviation (SD) and compared using two-sided *t*-tests. Ordinal data (e.g., Macnab score) were analyzed with the Mann–Whitney U test. Categorical variables were compared with χ^2^ or Fisher’s exact test, as appropriate. For dichotomous outcomes, odds ratios (OR) with 95% confidence intervals (CI) were calculated, using the conventional group as reference. For the primary endpoint (accuracy of screw placement), we additionally calculated the absolute risk reduction (ARR) and the number needed to treat (NNT). For continuous outcomes (estimated blood loss [EBL], length of stay [LOS], radiation time, VAS), results are reported as mean differences with 95% CI. A continuity correction of 0.5 was applied when one group had zero events. Statistical significance was set at *p* < 0.05. Analyses were performed with IBM SPSS Statistics, Version 27.0 (IBM Corp., Armonk, NY, USA). In addition, a post hoc power analysis was carried out for the primary endpoint (accuracy of screw placement, optimal vs. suboptimal) using Cohen’s h for two independent proportions with α = 0.05 (two-sided).

## 3. Results

### 3.1. Participants

A total of 240 patients were initially screened for eligibility. After reviewing medical records, 22 patients were excluded due to missing follow-up data or failure to meet the inclusion criteria, such as not undergoing pedicle screw placement because of alternative medical interventions or incomplete surgical data. This resulted in a final cohort of 218 patients, of whom 105 underwent robot-assisted pedicle screw placement, and 113 underwent conventional fluoroscopy-guided screw placement. All 218 patients completed follow-up and were included in the final analysis.

### 3.2. Baseline Characteristics

The baseline characteristics of the study participants, including demographic, clinical, and surgical data, are presented in Table 1. The mean age of participants was 67 years (±13), and the majority were female, comprising 58% (n = 61) of the robot-assisted group and 66% (n = 75) of the conventional group. The mean body mass index (BMI) was significantly higher in the robot-assisted group (28.69 ± 5.017) compared to the conventional group (26.75 ± 4.096). The primary surgical indications included degenerative disk disease, spinal canal stenosis, spondylolisthesis, and vertebral fractures. No significant differences were observed between the groups regarding the distribution of surgical indications (*p* > 0.05). Further details on patient characteristics, including comorbidities and preoperative status, are outlined in Table 1.

There were no missing data for the primary outcome of pedicle screw placement accuracy. Additionally, all 218 patients had complete data for other variables, including EBL, LOS, and postoperative complications.

### 3.3. Primary Outcome: Accuracy of Pedicle Screw Placement

**Figure 1:**
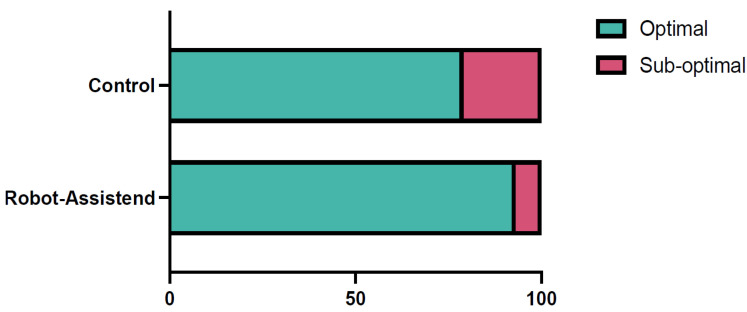
A bar diagram showing the accuracy of pedicle screw placement in the two cohorts. In the robot-assisted cohort optimal pedicle screw placement (Gertzbein–Robbins 0) was achieved in 93.33% of cases (n = 98/105), whereas in control cohort optimal placement was achieved in 78.76% of the cases (n = 89/113; OR = 3.78, 95% CI: 1.55–9.19, *p* = 0.003).

The accuracy of pedicle screw placement, assessed using the Gertzbein–Robbins classification, was significantly higher in the robot-assisted group compared to the conventional group. In the robot-assisted cohort, 93.33% of patients received a perfect screw placement (n = 98), classified as Grade 0 (optimal placement), compared to 78.76% (n = 89) in the conventional group (OR = 3.78, 95% CI: 1.55–9.19, *p* = 0.003) (Figure 1). The absolute risk reduction was 14.6%, and the number needed to treat (NNT) to achieve a Grade 0 screw placement with robot assistance was 6.9 (95% CI: 4.2–18.2). This indicates that approximately seven patients need to undergo robot-assisted surgery to prevent one suboptimal screw placement (Grade 1 or higher). Nevertheless, revision surgery due to screw misplacement was not significantly higher in the control cohort (4 (3.54%) in comparison to 2 (1.9%); *p* = 0.684). Additionally, an analysis based on the placement per screw revealed comparable trends as mentioned in Table 2. The post hoc power analysis for the primary endpoint (accuracy of screw placement, optimal vs. suboptimal) yielded a statistical power of 89.5% (effect size Cohen’s h = 0.436, α = 0.05, two-sided), confirming that the sample size was adequate to detect the observed between-group difference.

### 3.4. Secondary Outcomes

The robot-assisted group demonstrated significantly lower estimated blood loss (EBL) compared to the conventional group. The mean EBL in the robot-assisted group was 156.43 ± 102.22 mL, significantly lower than the 563.72 ± 280.48 mL observed in the conventional group (*p* < 0.001), with a large effect size (Cohen’s *d* = 1.93). Radiation exposure time was also significantly reduced in the robot-assisted group, with a mean of 109.27 ± 45.01 s compared to 239.88 ± 103.17 s in the conventional group (*p* < 0.001), demonstrating another large effect size (Cohen’s *d* = 1.64). However, mean surgery time did not differ significantly between the groups.

**Figure 2:**
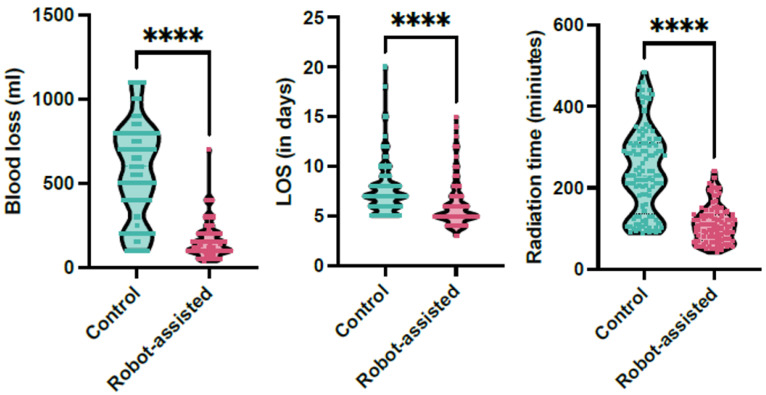
The whisker plots demonstrate the significant reduction in radiation time, length of hospital stay (LOS), and estimated blood loss in the robotic-assisted cohort (magenta) in comparison to the control cohort (green, **** signifies *p* < 0.001).

The length of hospital stay (LOS) was notably shorter in the robot-assisted group (6.12 ± 0.7 days) compared to the conventional group (7.6 ± 2.59 days; *p* < 0.0001, Figure 2). Postoperative pain, measured using the Visual Analog Scale (VAS), was significantly lower in the robot-assisted group (2.73 ± 0.72) than in the conventional group (2.94 ± 0.67); the mean difference was −0.21 (95% CI −0.395 to −0.025, *p* = 0.013), which is probably not clinically relevant.

Patient-reported outcomes based on the Macnab classification showed that a higher proportion of patients in the robot-assisted group achieved “Excellent” or “Good” results (Macnab grades 4–5: 73.33%) compared to the conventional group (45.13%; OR = 3.34, 95% CI: 1.89–5.91, *p* < 0.001). Conversely, “Fair” or “Poor” outcomes (Macnab grades 1–3) were more frequent in the conventional group (54.87%) than in the robot-assisted group (29.52%).

**Figure 3:**
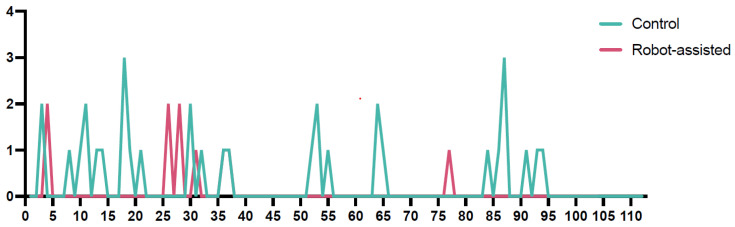
The run chart illustrates the accuracy of screw placement according to the Gertzbein–Robbins classification over time (*Y*-axis), comparing the conventional and robot-assisted cohorts. The green line represents the control cohort, while the continuous magenta line represents the robot-assisted cohort. Notably, in the robot-assisted cohort, there was a marked improvement in accuracy over time, with only one suboptimal screw placement occurring after the 40th procedure. In contrast, the accuracy in the conventional cohort did not show a significant improvement over time, maintaining a relatively stable rate of suboptimal placements throughout the study period. This highlights a potential learning curve benefit associated with the robot-assisted technique.

An interesting finding was that the accuracy of screw placement in the robot-assisted cohort improved over time. Most suboptimal placements occurred within the first 40 procedures. Afterwards, only one case with a Gertzbein–Robbins Grade 1 placement was observed (Figure 3).

### 3.5. Postoperative Complications

Postoperative complications and adverse events were generally similar between the robot-assisted and conventional groups, with comparable rates of revision surgeries, screw misplacements, and other adverse events, such as cerebrospinal fluid leaks and hematomas. Although surgical site infections were reported only in the robot-assisted group, this difference was not statistically significant. Overall, the complication profiles for both techniques appeared consistent. Further details of patients’ outcomes are outlined in Table 2.

## 4. Discussion

### 4.1. Accuracy of Pedicle Screw Placement

This study aimed to compare the accuracy and clinical outcomes of robot-assisted versus conventional fluoroscopy-guided pedicle screw placement in spinal surgery. The results demonstrated that robot-assisted techniques led to significantly higher accuracy rates in pedicle screw placement, as measured by the Gertzbein–Robbins classification, with 93.33% of screws in the robot group achieving an optimal placement (Grade 0) compared to 78.76% in the conventional group. The corresponding odds ratio (OR) was 3.78 (95% CI: 1.55–9.19), indicating that robot-assisted surgery was almost four times more likely to result in optimal screw placement. The absolute risk reduction was 14.6%, corresponding to a number needed to treat (NNT) of 6.9. These results are consistent with previous systematic reviews showing that robot-assisted techniques improve pedicle screw placement accuracy [3,4]. For example, the systematic review and meta-analysis of 19 studies by Fatima et al. found that robotic techniques significantly reduced misplacement rates compared to freehand methods, reinforcing the benefits we observed in our study [3].

These findings have also been corroborated by recent large-scale analyses. For example, Asada et al. assessed more than 1200 pedicle screws and confirmed superior accuracy with robotic systems, while Chumnanvej et al. reported in an updated meta-analysis that robotic assistance significantly improved accuracy and reduced radiation exposure compared to freehand placement [18,19].

Beyond accuracy, robotic assistance standardizes pedicle screw placement, reduces inter-operator variability [8], and provides valuable support to less experienced surgeons [9]. Although our study exclusively employed the Mazor X™ Stealth Edition (Medtronic, Minneapolis, MN, USA), recent meta-analyses comparing different platforms (Mazor, ExcelsiusGPS, ROSA, Cirq, TiRobot) have reported no significant differences in screw accuracy, with weighted accuracies consistently around 98% for Mazor, ExcelsiusGPS, and ROSA, and slightly lower for Cirq [20,21]. These results suggest that the improvements we observed are not limited to one platform but are generalizable across current robotic technologies.

It should be noted that the mean BMI was significantly higher in the robotic cohort (*p* = 0.002). A higher BMI is known to increase the technical challenge of fluoroscopy-guided pedicle screw placement, and this imbalance may have biased outcomes in favor of the robotic group. This should therefore be considered a potential confounder.

Previous studies further support the superiority of robotic systems. Li et al. demonstrated that both robot-assisted and navigation-assisted techniques significantly improve accuracy and safety of pedicle screw placement compared to freehand methods; however, the robotic systems showed superior precision over navigation-based approaches in scoliosis surgery. A systematic review further confirmed the enhanced accuracy and safety of robot-assisted techniques, underscoring their clinical advantages over navigation [22,23].

An additional noteworthy finding from this study was the observed increase in accuracy over time in the robotic cohort, with most suboptimal placements occurring within the first 40 procedures. After this initial phase, only one case with a Gertzbein–Robbins Grade 1 placement was recorded, indicating that as experience with the robotic system grew, placement accuracy reached consistently high levels. This learning curve effect suggests that proficiency with robotic systems can further optimize accuracy and underscores the importance of experience and training in maximizing the benefits of robotic-assisted surgery.

A study by Torii et al. analyzed the learning curve of robotic-assisted pedicle screw placement by comparing the performance of junior and experienced surgeons. The findings revealed that experienced surgeons achieved a plateau in accuracy after 25 cases, while junior surgeons required approximately 40 cases to reach similar levels of accuracy. Additionally, regression analysis indicated that significant reductions in surgical time and radiation exposure were observed after 30 cases, highlighting the steep learning curve associated with robotic systems. These results emphasize the necessity of adequate training and case volume to optimize the outcomes of robotic-assisted spinal procedures [9]. Similar results were also demonstrated in a trial published by Han et al. [24].

### 4.2. Length of Hospital Stay (LOS), Estimated Blood Loss (EBL), and Radiation Time

In our study, robot-assisted pedicle screw placement resulted in significantly lower estimated blood loss (EBL) and shorter length of hospital stay (LOS) compared to conventional techniques, findings that are consistent with prior research. Asada et al., in their analysis of 1633 lumbar fusion patients, observed that the robot-navigated group experienced significantly reduced EBL and LOS, with no substantial increase in operative time or reoperation rates [17]. This suggests that robotic guidance contributes to more efficient and minimally invasive procedures without compromising safety. Similarly, Li et al., through a meta-analysis, found that robot-assisted techniques consistently reduced intraoperative blood loss and hospitalization duration [25]. This reduction in EBL and shorter recovery times may be attributed to the increased precision and control offered by robotic systems, which minimize tissue disruption and optimize surgical trajectories.

In addition, Mason et al. highlighted that advanced imaging systems, such as 3D fluoroscopic navigation, improve screw placement accuracy, which indirectly reduces EBL and speeds up recovery. Their findings suggest that the accuracy provided by these imaging systems reduces intraoperative complications, thereby improving postoperative outcomes like LOS [26].

Furthermore, robot-assisted pedicle screw placement resulted in a significantly reduced radiation time in the operation room. This is in line with previous reports from Lin et al. that observed an inverse correlation of radiation time and experience with robotic surgery [13]. Reducing the radiation time during surgery is essential to minimize the cumulative dose received by employees during their work. Novel advancements like MRI-based 3D registration and augmented reality integration represent promising steps toward radiation-free yet highly accurate robotic surgery, enabling enhanced visualization and safety [27,28].

### 4.3. Clinical Outcomes

Furthermore, the result of this study regarding clinical outcomes favored the robot-assisted group, showing lower postoperative VAS pain scores and a higher percentage of patients achieving “Excellent” or “Good” outcomes on the Macnab classification. The odds of achieving an ‘Excellent’ or ‘Good’ Macnab outcome were more than tripled in the robot-assisted group compared to the conventional group (OR = 3.34, 95% CI: 1.89–5.91). Although the difference in VAS scores was statistically significant (Δ −0.21, 95% CI: −0.40 to −0.02), the clinical relevance of such a small effect size is limited. The improved pain and functional outcomes in the robotic cohort may partially reflect the precision of screw placement, which optimizes spinal stability and can reduce postoperative discomfort, a benefit noted in Li et al., whose meta-analysis reported that robot-assisted procedures resulted in improved patient-reported outcomes such as VAS and Oswestry Disability Index (ODI) scores, likely due to reduced intraoperative trauma and optimized screw placement [25].

### 4.4. Complications and Adverse Events

The occurrence of adverse events, such as revision surgeries, screw misplacements, CSF leaks, and hematomas, was comparable between the robot-assisted and conventional groups, indicating similar safety profiles. This aligns with Asada et al., who reported no significant differences in reoperation or readmission rates between techniques [18]. In patients with vertebral fractures, screw design and augmentation strategies may also influence fixation strength and outcomes. In particular, fenestrated or expandable screws augmented with polymethylmethacrylate (PMMA) have been shown to enhance stability in osteoporotic bone [29,30], highlighting the importance of precise screw placement in this setting. Such precision allows the use of the largest possible screw diameter within the pedicle and an optimal trajectory, thereby reducing the risk of loosening and related complications. Although robotic assistance may provide advantages in this regard, our data do not specifically address osteoporosis due to the lack of stratification and limited follow-up for long-term fixation outcomes. It should also be noted that osteoporosis itself has been identified as an independent risk factor for screw misplacement not only in robot-assisted surgery [31,32] but also in conventional fluoroscopy-guided techniques [33]. Furthermore, Mason et al. indicated that advanced image guidance, such as 3D fluoroscopic navigation, can help enhance screw accuracy and minimize intraoperative errors, indirectly contributing to favorable clinical outcomes and comparable safety between techniques [26]. Marcus et al. reviewed five studies and found some support for fluoroscopy-guided techniques in terms of lower infection and adverse event rates [5]. However, the high precision of robotic systems in preventing screw misplacement may outweigh the minor increases in complications like infections and hematomas, especially given that our study and others report lower overall complication rates with robotic systems [3,4,5].

### 4.5. Effect on Operating Time

Operating times were comparable between the robot-assisted and conventional groups, suggesting that proficiency mitigates robotic setup and calibration delays. This is consistent with findings from Li et al., Asada et al., and Han et al., which showed no significant increases in operative times with robotic surgery [18,22,24]. However, our findings contrast with those of Fatima et al. and Gao et al., both meta-analyses that reported a notable increase in operative times for robot-assisted procedures—approximately 20.5 and 22.7 min longer, respectively. The prolonged duration in these studies is often attributed to the initial setup and calibration requirements unique to robotic systems, which may introduce more complexity compared to the straightforward preparation needed for freehand techniques [3,4].

### 4.6. Future Prospects: Enhanced Preoperative Planning with AI and Personalized Treatment

Robotic precision in pedicle screw placement supports advanced preoperative planning, with AI poised to optimize trajectories based on patient-specific anatomy. This innovation could enhance accuracy, reduce complications, and minimize revisions. The integration of AI into robotic platforms represents a critical avenue for research, advancing individualized treatment approaches [34,35].

### 4.7. Cost-Effectiveness

Despite concerns over the high costs of robotic spine surgery, a study by Menger et al. supports its cost-effectiveness, citing reduced complications, fewer infections, and shorter hospital stays. Improved pedicle screw accuracy alone could prevent approximately 9.47 revision surgeries annually, saving $314,661. Additionally, converting open surgeries to minimally invasive procedures saved $608,546 annually in a single center [36]. As AI integrates into robotic systems, the potential for personalized, efficient, and cost-effective spinal surgeries increases, further addressing concerns about costs while enhancing precision and patient outcomes.

### 4.8. Limitations

Several limitations must be considered when interpreting the results of this study. First, as a single-center retrospective cohort study, the findings may be subject to selection bias and residual confounding. Allocation to robotic or conventional treatment depended on logistical availability and surgeon preference, rather than randomization. This introduces potential selection bias, as unmeasured factors such as case complexity or anatomical variability may have influenced both allocation and outcomes. Although variables like preoperative VAS scores and BMI were recognized as potential confounders, they were not fully adjusted due to the study’s exploratory scope and sample size limitations. Future studies with larger samples and multi-center designs should incorporate comprehensive adjustments for these confounding variables to confirm the findings.

Additionally, the relatively small sample size may limit the generalizability of these results. Moreover, long-term outcomes, such as screw loosening or spinal stability over time, were not addressed in this study, which restricts our ability to draw conclusions on the long-term efficacy of robot-assisted techniques. Imprecision in measurements, such as intraoperative blood loss estimation, may have introduced variability. Infection rates were noted, but there was no standardized postoperative infection control protocol across all patients, which may have influenced results.

Lastly, the high cost and required training for robotic systems could limit accessibility, particularly in smaller facilities. Financial constraints and the investment in training and infrastructure needed to proficiently use robotic systems must be weighed against the potential improvements in patient outcomes [37]. The patient population in this study consisted primarily of individuals with degenerative spinal conditions and vertebral fractures, which may not fully represent other demographics. Consequently, results may not be universally applicable, especially in settings with varied patient profiles or healthcare resources. Institutions lacking access to robotic technology or trained teams may not experience the same benefits observed in this study. Future research across multiple centers is needed to validate these findings in diverse healthcare settings.

## 5. Conclusions

This retrospective cohort study demonstrates that robot-assisted pedicle screw placement improves the accuracy of screw placement and reduces intraoperative blood loss and hospital stays compared to conventional fluoroscopy-guided techniques. These results add to the available evidence supporting the benefits of robotic assistance in spinal surgery, reinforcing findings from previous research on its precision and efficiency. While robotic systems provide significant short-term benefits in spinal surgery, future research should focus on long-term outcomes, cost-effectiveness, and the wider generalizability of these results. As surgical technologies continue to evolve, robot-assisted techniques are likely to play an increasingly important role in enhancing surgical precision and patient care.

## Figures and Tables

**Table 1:** Demographic Characteristics.

Variable	Robot-Assisted (n = 105)	Control Group (n = 113)	*p*-Value (Statistical Test Used)
**Sex**			0.212 (Fisher’s Exact)
**Female (n, %)**	61 (58.1%)	75 (66.4%)	
**Male (n, %)**	44 (41.9%)	38 (33.6%)	
**Age (Mean, SD)**	64.72 (±13.13)	66 (±13.28)	0.873 (two-tailed *t*-test)
**BMI (Mean, SD)**	28.69 (±5.017)	26.75 (±4.096)	0.002 (two-tailed *t*-test)
**Repeated surgery (n, %)**	36 (34.29%)	43 (38.05%)	0.576 (Fisher’s Exact)
**VAS pre-OP (Mean, SD)**	7.72 (±0.7)	7.6 (±0.59)	0.1647 (Two-tailed *t*-test)
**Indication for surgery**			0.628 (χ^2^-test)
**Degenerative Disk Disease (n, %)**	31 (29.52%)	34 (30.08%)	
**Spinal canal stenosis (n, %)**	19 (18.09%)	24 (21.23%)	
**Spondylolisthesis (n, %)**	23 (21.90%)	29 (25.66%)	
**Fracture (n, %)**	32 (30.47%)	26 (23.00%)	
**ASA Score**			0.404 (χ^2^-test)
**1 (n, %)**	9 (8.6%)	20 (17.70%)	
**2 (n, %)**	53 (50.48%)	20 (17.70%)	
**3 (n, %)**	32 (30.48%)	34 (30.09%)	
**4 (n, %)**	1 (0.95%)	1 (0.88%)	

ASA: American Society of Anaesthesiologists Physical Status Classification System; BMI: Body Mass Index; M/F: Male/Female; SD: Standard Deviation; OP: Operation; VAS: Visual Analog Scale.

**Table 2:** Postoperative patient outcomes.

Variable	Robot-Assisted (n = 105)	Control Group (n = 113)	*p*-Value (Statistical Test Used)
**Estimated blood loss** **(ml, Mean, SD)**	156.43 (±102.22)	563.72 (±280.48)	<0.001 (Two-tailed *t*-test)
**Radiation time (Sec, Mean, SD)**	109.27 (±45.01)	239.88 (±103.17)	<0.001 (Two-tailed *t*-test)
**Surgery time (min, Mean, SD)**	221.51 (±86.73)	236.93 (±94.69)	0.212 (Two-tailed *t*-test)
**LOS (days, Mean, SD)**	6.12 (±0.7)	7.6 (±2.59)	<0.001 (Two-tailed *t*-test)
**VAS post-OP**	2.73 (±0.72)	2.94 (±0.67)	0.013 (Two-tailed *t*-test)
**Number of screws implanted per patient (Sum)**	508	570	0.698 (χ^2^-test)
**Four (n, %)**	71 (67.62%)	68 (60.18%)	
**Six (n, %)**	25 (23.81%)	32 (28.32%)	
**Eight (n, %)**	8 (7.19%)	12 (10.62%)	
**Ten (n, %)**	1 (0.95%)	1 (0.88%)	
**Gertzbein–Robbins Classification per patient**			0.029 (χ^2^-test)
**0 (n, %)**	98 (93.33%)	89 (78.76%)	
**1 (n, %)**	4 (3.81%)	17 (15.04%)	
**2 (n, %)**	3 (2.85%)	5 (4.42%)	
**3 (n, %)**	0	2 (1.77%)	
**Gertzbein–Robbins** **dichotomic per patient**			0.003 (Fisher’s Exact) OR = 3.78 (95% CI: 1.55–9.19)
**Optimal (0) (n, %)**	98 (93.33%)	89 (78.76%)	
**Suboptimal (1–3) (n, %)**	7 (6.66%)	24 (21.24%)	
**Gertzbein–Robbins dichotomic, per screw**			0.007 (Fisher’s exact) OR = 2.76 (95% CI: 1.28–5.92)
**Optimal (0) (n, %)**	499 (98.23%)	543 (95.26%)	
**Suboptimal (1–3) (n, %)**	9 (1.77%)	27 (4.74%)	
**Postoperative Macnab (points, Median, IQR)**	4 (3–4)	3 (3–4)	<0.001 (MWU)
**5 (n, %)**	14 (13.33%)	3 (2.65%)	
**4 (n, %)**	63 (59.05%)	48 (42.48%)	
**3 (n, %)**	21 (20%)	57 (50.44%)	
**2 (n, %)**	7 (6.67%)	3 (2.65%)	
**1 (n, %)**	0	2 (1.77%)	
**Macnab 4–5 (n, %)**	77 (73.33%)	51 (45.13%)	<0.001 (Fisher’s Exact) OR = 3.34 (95% CI: 1.89–5.91)
**Macnab 1–3 (n, %)**	28 (26.66%)	62 (54.87%)	
**Revision Surgery (n, %)**	7 (6.67%)	7 (6.19%)	>0.99 (Fisher’s Exact)
**Screw misplacement (n, %)**	2 (1.9%)	4 (3.54%)	0.684 (Fisher’s Exact)
**Surgical site infection (n, %)**	2 (1.9%)	0	>0.99 (Fisher’s Exact)
**CSF leak (n, %)**	2 (1.9%)	2 (1.77%)	>0.99 (Fisher’s Exact)
**Hematoma** (**n, %)**	1 (0.95%)	1 (0.88%)	>0.99 (Fisher’s Exact)
**Medical Adverse events (n, %)**	2 (1.9%)	3 (2.6%)	>0.99 (Fisher’s Exact)

CI: Confidence Interval; CSF: Cerebrospinal fluid; IQR: Interquartile Range; LOS: Length of Stay; MWU: Mann–Whitney U test; OP: Operation; OR: Odds ratio; SD: Standard Deviation; VAS: Visual Analog Scale.

## Data Availability

Summary data supporting the findings of this study are included in the article. Individual patient-level data are not publicly available due to privacy and ethical restrictions but may be made available from the corresponding author upon reasonable request.

## Abbreviations

The following abbreviations are used in this manuscript:

ASA	American Society of Anaesthesiologists Physical Status Classification System
BMI	Body Mass Index
CI	Confidence Interval
CSF	Cerebrospinal Fluid
CT	Computed Tomography
EBL	Estimated Blood Loss
IRB	Institutional Review Board
IQR	Interquartile Range
LOS	Length of Stay
MWU	Mann–Whitney U Test
NNT	Number Needed to Treat
ODI	Oswestry Disability Index
OR	Odds Ratio
RR	Relative Risk
SD	Standard Deviation
STROBE	Strengthening the Reporting of Observational Studies in Epidemiology
